# Cervical screening: the evolving landscape

**DOI:** 10.3399/bjgp22X720197

**Published:** 2022-07-29

**Authors:** Jennifer C Davies-Oliveira, Thomas Round, Emma J Crosbie

**Affiliations:** Gynaecological Oncology Research Group, Division of Cancer Sciences, University of Manchester; Department of Obstetrics and Gynaecology, St Mary’s Hospital, Manchester University NHS Foundation Trust, Manchester Academic Health Science Centre, Manchester.; Population Health Sciences, King’s College London, London.; Gynaecological Oncology Research Group, Division of Cancer Sciences, University of Manchester; Department of Obstetrics and Gynaecology, St Mary’s Hospital, Manchester University NHS Foundation Trust, Manchester Academic Health Science Centre, Manchester.

The elimination of cervical cancer is within reach. Together, vaccination and screening are so effective at preventing the sequelae of persistent high-risk human papillomavirus (hr-HPV) infection that the World Health Organization has set ambitious targets for their global implementation by 2030, in an attempt to achieve this goal.^1^ Real world data from the UK show that vaccines that trigger immunity against hr-HPV are 87% effective at reducing cervical cancer risk if deployed in the early teen years.^2^ The NHS Cervical Screening Programme has reduced deaths from cervical cancer by up to 70% since its introduction over three decades ago.^3^ The discovery that HPV is the causative organism coupled with high throughput technology to detect it has enabled hr-HPV testing to replace cytology as the primary test in cervical screening, with superior sensitivity for cervical precancer detection the major advantage.^4^

Yet uptake of screening in the UK is low and falling. The most recent figures suggest that only 70.2% of those eligible are up-to-date with screening.^5^ The COVID-19 pandemic has contributed further to this decline.^6^ Screening uptake shows geographic disparities and is particularly low in those aged <30 and >50 years of age, in people of non-White British ethnicities, those who identify as LGBTQIA+, and in socioeconomically deprived communities.^7^^,^^8^ The main barriers to screening are finding time to attend an appointment in primary care during working hours and the embarrassment and discomfort of the speculum examination.

## TACKLING POOR UPTAKE

Removing barriers is key to increasing participation, which has the potential to further reduce the UK incidence of cervical cancer by up to 13%.^3^ Various strategies to improve participation have been tried; however, non-speculum sampling options show greatest promise.^9^ Non-speculum sampling capitalises on the physiology of hr-HPV-infected cervical cells shedding through the lower genital tract, from where they can be collected with a vaginal swab or in the first fraction of voided urine as a flushed contaminant.

## VAGINAL SELF-SAMPLING

The non-inferior test accuracy^10^ of vaginal sampling for hr-HPV detection has prompted nine countries to adopt it as their primary cervical screening collection method,^11^ and more will certainly follow, including Australia from July 2022. Growing evidence shows preference for self-sampling, with a recent survey suggesting 51.4% of UK invitees and over 70% of imperfect or never attenders would choose self-screening.^12^ Large pilot studies for population-based vaginal self-sampling, YouScreen and HPValidate, are underway to confirm the feasibility of national implementation, and their results are eagerly awaited. Self-sampling enables the convenience and privacy of home-based collection, reducing pressure on overburdened NHS primary care services. The practicalities of offering vaginal self-sampling need to be understood and learning sought from other programmes where this method has been adopted.

Despite its proven non-inferiority to routine cervical screening, adequate vaginal self-sample acquisition remains a user concern. To address this concern, Landy *et al* explored the diagnostic performance and acceptability of non-speculum clinician obtained vaginal samples compared to routine cervical screening in primary care and colposcopy clinic attendees. Published in this issue of the *BJGP*, their results suggest good diagnostic accuracy for cervical precancer detection using non-speculum clinician sampling and high acceptability.^13^ Previously published in this journal, the same group explored uptake of non-speculum clinician sampling for those aged ≥50 years who were late for screening by ≥12 months.^14^ Targeting this age group is deliberate and important as individuals aged >65 years account for around half of all cervical cancer deaths.^15^ Those inadequately screened prior to exiting the NHS Cervical Screening Programme and/or with unknown hr-HPV status are most at risk, prompting some countries to extend the screening age to 75 years or offer a one-off ‘catch up’ hr-HPV test.^16^ Self-sampling and non-speculum clinician sampling options increased the absolute uptake of screening by 17% compared to the control group, with just over half opting for non-speculum clinician obtained (22.5%) and self-sampling (35.8%) options.^13^ Non-speculum sampling could be offered as an empowering educational event to enable future home-based self-sampling. Further exploration in adequately powered studies with sufficient cases of cervical precancer to confirm clinical test accuracy and acceptability are now required.

## URINE SELF-SAMPLING

Another potential sample type for cervical screening is urine, which has advantages in terms of familiarity with self-collection and avoidance of an intimate procedure. Promising diagnostic accuracy for hr-HPV detection^17^ and acceptability with users has been reported when urine is collected using a bespoke first void urine collection device called the Colli-Pee (Novosanis, Wijnegem, Belgium). The ACES (Alternative Cervical Screening) suite of studies in Manchester, UK, will establish with precision how urine compares to cervical sampling for cervical precancer detection, and provide proof of principle for home-based urine self-collection with postal return for hr-HPV testing in current cervical screening non-attenders.

## EMBEDDING NEW APPROACHES IN CLINICAL PRACTICE

Despite recent advances in non-speculum sampling options for improving uptake of cervical screening, we must proceed with caution before discarding routine cervical screening methodology altogether. Further exploration of acceptability is needed given the differences in preference highlighted by Landy *et al* among different ethnic groups^13^ and those with abnormal screening results (ACES Colposcopy Study, unpublished data). Choice is important, and a menu of screening options would seem the most pragmatic way of ensuring high uptake and satisfaction. Self-sampling is likely to be more cost-effective for a resource-limited NHS, and this must be taken into consideration when shaping the future direction of the Cervical Screening Programme. Healthcare provider and public education campaigns will be essential when implementing changes, as highlighted by the recent negative public reaction to changing the screening interval in Wales from 3- to 5-yearly, despite strong scientific evidence for its safety.^18^

While ∼87% of those screened are negative for hr-HPV and need no further intervention, those who are positive present a quandary. Reflex cytology is not available for non-cervical sampling methods and, therefore, a repeat sample collected from the cervix is offered to collect this information. Encouragingly, 80%–90% of individuals with a positive self-sample state that they would attend for further investigations.^19^ A more streamlined approach would be to subject the hr-HPV positive sample to molecular triage, for example, by extended genotyping or methylation analysis, to distinguish those who need immediate colposcopy referral from those who can be safely deferred for a year.^20^^,^^21^ Emerging evidence suggests that methylation biomarkers may be effective means of molecular triage irrespective of sample type,^22^ and several rival tests are on the horizon.

## CONCLUSION

The cervical screening landscape is evolving rapidly, and alongside its vaccine ally, brings with it an optimistic hope for the global elimination of cervical cancer. Non-speculum sampling approaches show huge promise for improving screening uptake and reducing the burden of disease. We must now focus on validating non-speculum sampling test accuracy, through YouScreen, HPValidate, and ACES studies, while determining its acceptability and feasibility for national implementation, being sure to cast the net as wide as possible to maximise its benefits.
